# Advancing Our Understanding of Pyranopterin-Dithiolene Contributions to Moco Enzyme Catalysis

**DOI:** 10.3390/molecules28227456

**Published:** 2023-11-07

**Authors:** Sharon J. Nieter Burgmayer, Martin L. Kirk

**Affiliations:** 1 Department of Chemistry, Bryn Mawr College, Bryn Mawr, PA 19010, USA; 2 Department of Chemistry and Chemical Biology, The University of New Mexico, Albuquerque, NM 87131, USA

**Keywords:** molybdenum enzymes, pyranopterin, molybdopterin, dithiolene, molybdenum cofactor, Moco

## Abstract

The pyranopterin dithiolene ligand is remarkable in terms of its geometric and electronic structure and is uniquely found in mononuclear molybdenum and tungsten enzymes. The pyranopterin dithiolene is found coordinated to the metal ion, deeply buried within the protein, and non-covalently attached to the protein via an extensive hydrogen bonding network that is enzyme-specific. However, the function of pyranopterin dithiolene in enzymatic catalysis has been difficult to determine. This focused account aims to provide an overview of what has been learned from the study of pyranopterin dithiolene model complexes of molybdenum and how these results relate to the enzyme systems. This work begins with a summary of what is known about the pyranopterin dithiolene ligand in the enzymes. We then introduce the development of inorganic small molecule complexes that model aspects of a coordinated pyranopterin dithiolene and discuss the results of detailed physical studies of the models by electronic absorption, resonance Raman, X-ray absorption and NMR spectroscopies, cyclic voltammetry, X-ray crystallography, and chemical reactivity.

## 1. Introduction

The pyranopterin molybdenum (Mo) enzymes factor prominently in global biogeochemical cycles and are critical to the life processes of most organisms on Earth [1,2,3,4,5,6,7]. In humans, these enzymes catalyze reactions that contribute to the production of reactive oxygen species associated with postischemic reperfusion injury [8,9] and oxidative stress [10], xenobiotic detoxification [10,11,12,13,14,15,16], drug metabolism [11,17,18,19,20,21,22,23,24] and prodrug activation [21,25], nitrite to NO conversion [24,26,27,28,29,30,31,32,33], sulfite oxidation [1,34,35,36], molybdenum cofactor (Moco) sulfuration [26,33,37,38,39,40,41,42], and amino acid catabolism [43]. The importance of these enzymes in humans is underscored by the fact that Moco deficiency can result in early infant mortality [24]. More recently, pyranopterin Mo enzymes have been found to play key roles in the gut microbiome [17,44,45,46] and as methionine sulfoxide reductases in respiratory pathogens (e.g., *Haemophilus influenzae*) [1,47,48,49,50]. These are unusual metalloenzymes since they employ second- (Mo) and third-row (W) transition metal ions to perform a myriad of two-electron redox transformations [4]. Furthermore, Moco in these molybdoenzymes is unique in possessing a pyranopterin dithiolene ligand (PDT; also known as molybdopterin) [37,51,52,53,54,55,56], and Moco is biosynthesized in a complex series of reactions by nine different gene products in bacteria and seven in plants and humans [37,39,54,55,56,57,58,59,60]. The related pyranopterin W enzymes possess a closely analogous cofactor, the tungsten cofactor, or Tuco [3,61,62].

**Figure 1:**
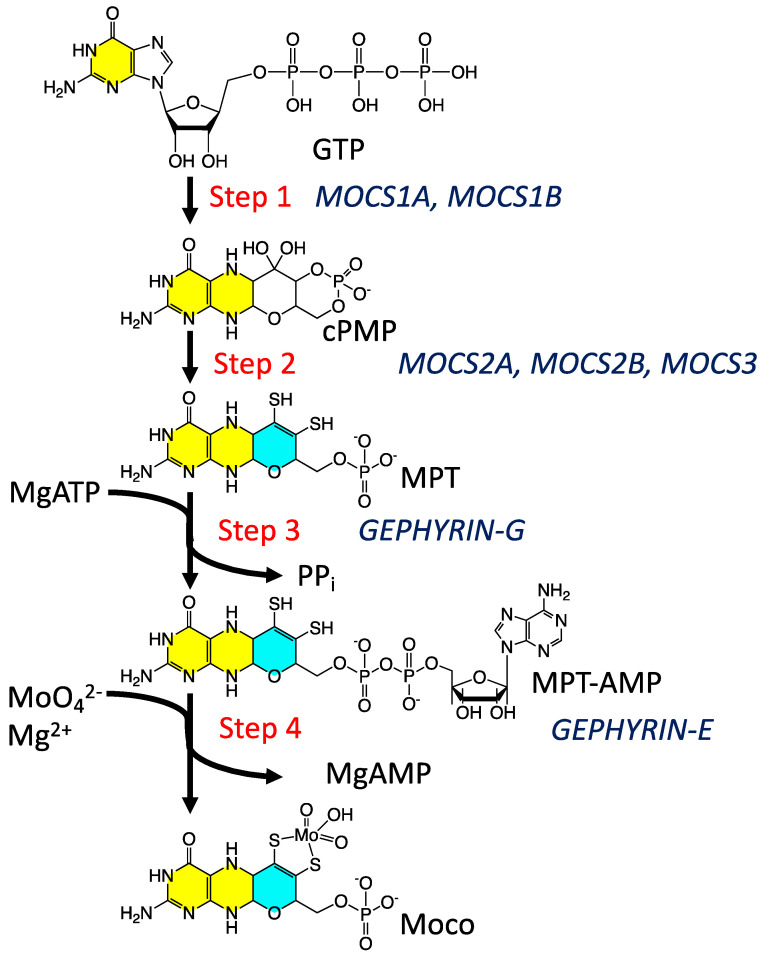
The Moco biosynthetic pathway. The two yellow-highlighted rings comprise the pterin structure, while the cyan ring is the adjoining pyran ring.

Moco biosynthesis [37] in plants and higher organisms is often described in terms of four fundamental steps, with an additional biosynthetic step in archaea and bacteria (Figure 1) [37,39,54,56,57,63,64]. Step 1 is the formation of a cyclic pyranopterin monophosphate from GTP. In the second and third steps, molybdopterin (MPT) is formed, and MPT is adenylated. Following adenylation, Mo derived from molybdate is inserted into MPT to form Moco. The fifth step in bacteria and archaea involves a further modification of Moco, where a nucleotide is attached to the MPT phosphate to form a dinucleotide version of Moco. The biosynthesized Moco is then inserted [55,57,60] into apo proteins that have traditionally been divided into three enzyme families [6,65] described as the xanthine dehydrogenase (XDH), sulfite oxidase (SUOX), and dimethyl sulfoxide reductase (DMSOR) families. Despite our emerging knowledge of the relationship between structure and function in these enzymes and our general understanding of enzyme-catalyzed oxygen atom transfer reactivity and the hydroxylation of N heterocycles, we know remarkably little about the role of the PDT in their catalytic cycles. While a large number of high-quality reviews have detailed aspects of cofactor biosynthesis [52,57,63,64,66], enzyme structure [1,6,13,67,68,69], reaction mechanisms [1,6,34,70,71,72], spectroscopy [5,34,71,73,74,75], and electronic structure contributions to reactivity [1,5,51,70,75], there has not been an account that has focused on our understanding of the PDT from the perspective of small molecule studies. Here, we detail recent discoveries regarding the nature of the PDT and how this complex, highly non-innocent biological ligand may affect the electronic structure of enzyme active sites to promote catalysis.

## 2. What Is Currently Known about Moco in the Enzymes?

Studies of the molybdenum cofactor span more than half a century. A succinct description of this history of Moco has been provided by Mendel in the recent *Molecules* series on the State of the Art in Molybdenum Cofactor Research [52]. Here we give a brief overview of the features of Moco that have guided recent research on model compounds in our laboratories.

### 2.1. Protein X-ray Crystallography Gives Atomic Level Views of Moco

**Figure 2:**
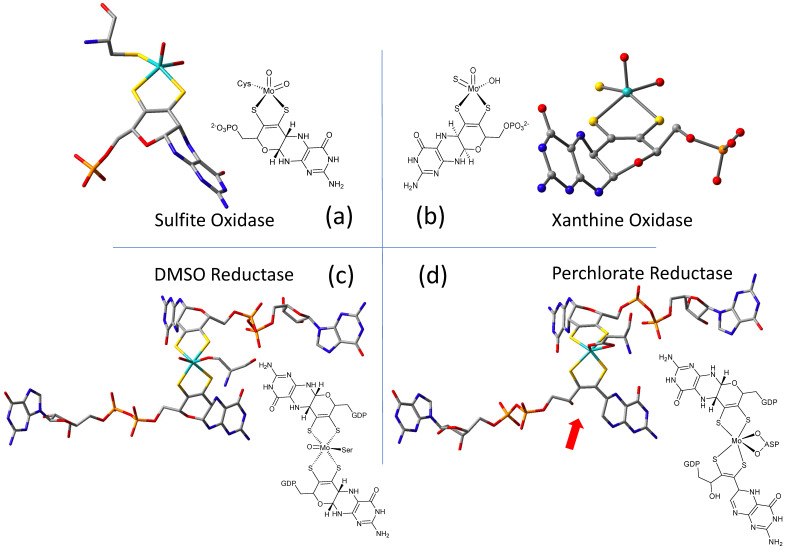
Representative examples of Moco structures for each of the SO, XO, and DMSO families, and one example of Moco possessing one PDT having an open, uncyclized pyran ligand. (**a**) Sulfite Oxidase (PDB 1SOX). (**b**) Xanthine Oxidase (PDB 3NRZ). (**c**) Dimethylsulfoxide Reductase (PDB 1EU1). (**d**) Perchlorate Reductase (PDB 5CH7) where the red arrow points to the open pyran ring position.

**Figure 3:**
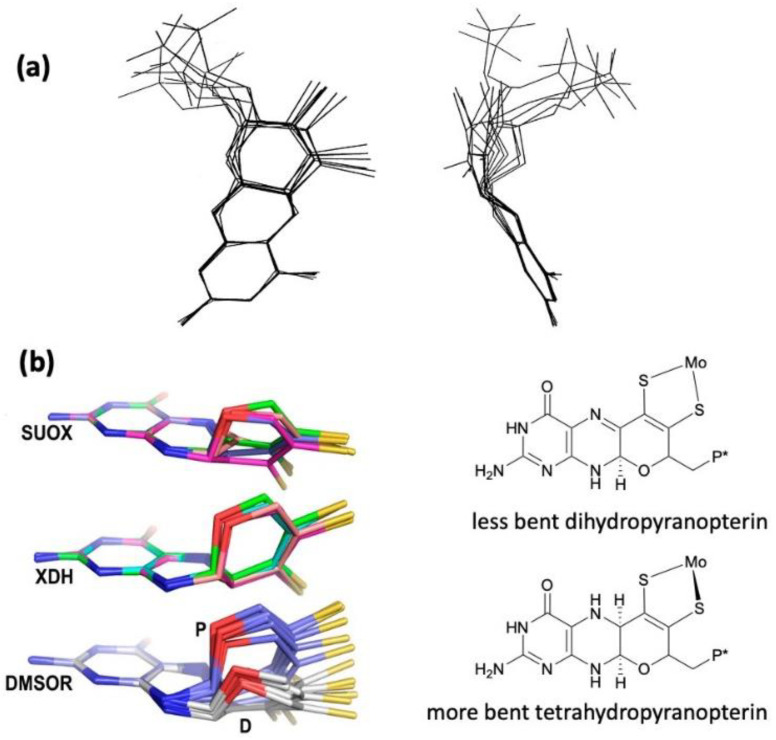
(**a**) Two views of the range of pyranopterin conformations observed in 1997. (**b**) Two distinct conformations suggest that pyranopterins in Moco have different oxidation states in different families. P* denotes a phosphate or a dinucleotide terminus. Adapted from Ref. [79].

The W-containing aldehyde ferredoxin oxidoreductase [76] and Mo DMSOR [77] were structurally characterized in 1995 and 1996, respectively, and the structures represent the first for any pyranopterin tungsten or molybdenum enzyme. Now there are crystal structures for a large number of molybdoenzymes, and this has led to a dramatic increase in our understanding of these enzymes. Figure 2 shows Moco as found in each of the three canonical molybdoenzyme families: sulfite oxidase (SUOX), xanthine oxidase (XDH), and dimethylsulfoxide reductase (DMSOR). These structures are depicted both as a three-dimensional image and in a bond line drawing representation. The 3D views emphasize the non-planar, bent nature of the pyranopterin component of Moco. As more examples of Moco structures located in different protein environments became available through protein crystallography, the dramatic range of pyranopterin conformations within the PDT ligand became apparent. This flexibility in pyranopterin conformation was noted as early as 1997 [78], and it is depicted as an overlay of the pyranopterin portions of Moco from different protein crystal structures (Figure 3a). A more recent analysis of the metrical differences in the folding of 319 pyranopterins in 102 molybdenum protein structures led to the identification of two main pyranopterin conformations observed in the protein structures and to the proposal that the pterin might have different oxidation states among the three families (Figure 3b) [79]. From this study emerged the proposal that the highly bent PDT ligands displayed in the XDH family enzymes corresponded to fully reduced pyranopterin structures, whereas the less bent pyranopterins in the PDTs from SUOX family proteins better fit a dihydropyranopterin structure (Figure 3b). Intriguingly, the two PDT pyranopterins in the DMSOR family of proteins exhibited different conformations, where the highly bent proximal pyranopterin fits a reduced pyranopterin description while the distal PDT ligand is less bent and consistent with a dihydropterin assignment.

A second type of structural anomaly is observed within the pyranopterin portion of Moco. Among the large number of molybdoenzyme structures, there are three proteins—all members of the DMSOR family—whose structures clearly show the distal PDT ligand with no pyran ring [80]. The first such example identified was dissimilatory nitrate reductase (NarGHI) from *E. coli* [81], followed by ethyl benzene dehydrogenase (EBDH) [82]. The most recent example is perchlorate reductase (PcrAB) [83] from *Azospira suillum,* which is shown as a representative example for this structural type in Figure 2d.

The above examples highlight how both the conformation of the pyranopterin portion of Moco and the presence of a pyran ring vary between enzyme families and even within a family. This has been interpreted to suggest that pterin modification is being manipulated to adjust the catalytic site reactivity as required by the organism.

**Figure 4:**
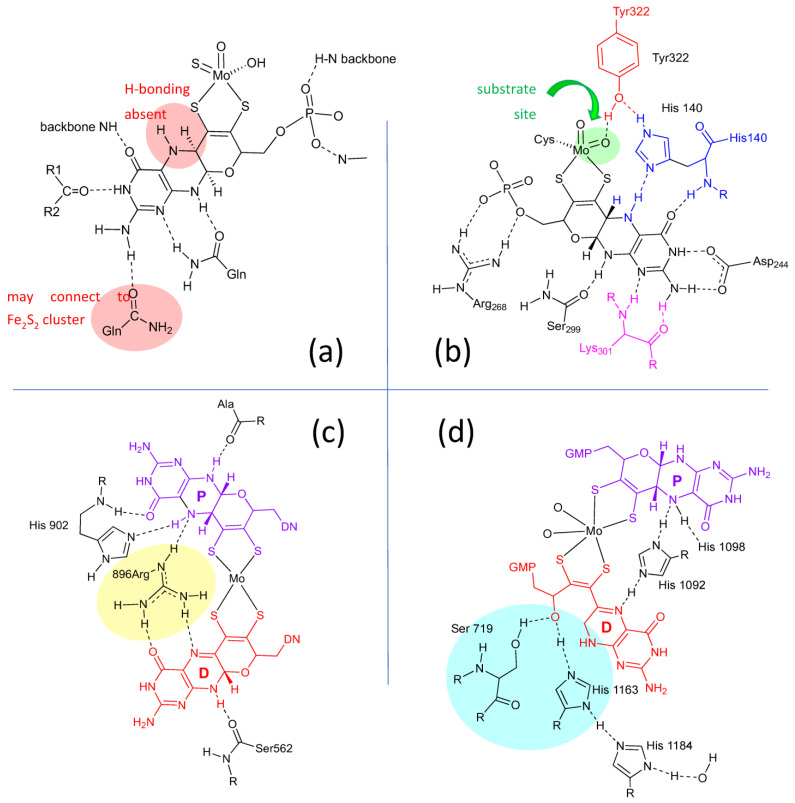
H-bonding interactions between the pyranopterin of Moco and adjacent protein residues. (**a**) Conserved H-bonding interactions identified for all 7 members of the XDH family. (**b**) Conserved H-bonding interactions identified for three members of the SUOX family. (**c**) H-bonding interactions within the DMSOR protein structure that are representative of 15 other members of the DMSOR family. (**d**) H-bonding interaction in *E. coli* nitrate reductase, whose Moco exhibits one non-cyclized pterin structure.

Lastly, the protein environment that encapsulates and protects Moco from degradation is recognized to play a role in Moco function. The abundance of H-bonds tethering pyranopterin to the protein is recognized to enforce the proper orientation and conformation of the cofactor. However, H-bonding analysis shows several other ways that H-bonds—or indeed, their absence—might be involved in catalysis. A study of all known PDT-containing protein structures that analyzed patterns of hydrogen bonding interactions between protein residues and Moco revealed multiple conserved features within each protein family [80]. These are summarized pictorially in Figure 4. Identical H-bonding is observed among seven proteins within the XDH family (Figure 4a), where protein backbone amide NH groups stabilize C=O and P=O bonds in PDT. Notable features in the XDH family are the conserved absence of H-bonding to pterin N5 and the H-bonding between the amino group at C8 and a glutamine residue that connects to an adjacent Fe_2_S_2_ cluster. A majority of proteins in the SUOX family exhibit a network of H-bonds linking the pterin N5 to the equatorial oxygen via histidine (blue) and tyrosine residues (red), as shown for SO in Figure 4b. The Tyr residue is conserved in sulfite oxidase proteins and is proposed to function as a charge transfer relay system [80], where tautomerization results in formal charge migration in the protein. In contrast, this Tyr residue is absent in nitrate reductase members of the SUOX family. H-bonding provided by Lys301 residue (Figure 4b, pink) is the site where a mutation to Arg corresponds to sulfite oxidase deficiency. In members of the DMSOR family (Figure 4c), a conserved arginine residue bridges the proximal pterin to the distal pterin via a network of H-bonds (yellow highlight) and is suggestive of important charge communication between the two pyranopterins (see Section 2.3.4). Proteins in all three families (SUOX, XDH, and DMSOR) typically lack any H-bonding in the region of the pyran ring oxygen atom. In contrast, the unusual uncyclized PDT in dissimilatory nitrate reductase [81] (Figure 4d) reveals several H-bonding interactions involving serine and histidine residues (Figure 4d, cyan highlight) that may assist in facilitating and maintaining the open conformation of the pyran ring. In conclusion, the structural evidence cited above points to the pyranopterin of Moco playing a viable role in catalysis, albeit one that has yet to be clearly defined. By contrast, there is ample evidence for how the dithiolene chelate may control and modulate the Mo redox chemistry [2,51,84,85,86,87,88,89,90,91,92], and its function as a component of Moco is already well established.

### 2.2. Information about the PDT of Moco Obtained from Spectroscopy

Direct spectroscopic studies that inform on the PDT component of Moco are sparse [89,90], and this is due to the fact that the vast majority of pyranopterin Mo enzymes possess additional highly absorbing chromophores, including flavin, [2Fe-2S], [4Fe-4S], and heme. However, spectroscopic probes of the PDT in molybdoproteins that lack these chromophores and in relevant model systems that possess a coordinated PDT ligand are important, and these studies will assist in defining both the proposed and still unknown role(s) of the PDT in catalysis. As detailed in the prior section, the PDT has no covalent interactions with the protein, and X-ray crystallography provides strong evidence that the PDT is extensively hydrogen bonded to the protein [80]. Furthermore, these hydrogen bonding interactions are different for different proteins, and they possess the potential to modulate both the geometric and electronic structure of the PDT in order to fine-tune the active site for specific substrate transformations and electron transfer reactivity.

Early resonance Raman spectroscopic studies were performed on *R. sphaeroides* and *R. capsulatus* DMSORs [93,94,95] and biotin sulfoxide reductase [96], but more direct probes of hydrogen bonding between the protein and the pyranopterin derive from Raman studies on bovine and bacterial XO/XDH [89,90,97,98]. These latter studies have been particularly revealing from the standpoint of observing low-frequency modes assignable to the PDT. Using lumazine as the reducing substrate, it has been shown that XO/XDH catalyzes the two-electron conversion of this substrate to violopterin [89,90,97,98,99,100,101,102], which subsequently binds strongly to the Mo(IV) center, allowing for spectral probing of an enzyme-product complex by optical spectroscopies. This Mo(IV)-violopterin state possesses an intense charge transfer band that absorbs light in the red/NIR region of the optical spectrum, providing an opportunity to probe a catalytically relevant product-bound species formed by enzymatic turnover by optically pumping into this band and probing the nature of resonantly enhanced protein and product vibrations. The importance of this band being in the red/NIR region of the spectrum is underscored by the fact that spectral contributions from both the [2Fe-2S] clusters and FAD are effectively eliminated, and this includes any notable background fluorescence from the FAD. The early resonance Raman studies by Hille and coworkers [98], which indicated that the low-energy charge transfer band was Mo → violapterin in nature, showed that numerous vibrational modes associated with the violopterin product were observed. The lower frequency vibrations in the 250–1100 cm^−1^ region were postulated to arise from the Mo coordination sphere. These studies suggested that the product was bound end-on to Mo(IV) in an Mo-O-R fashion [98].

**Figure 5:**
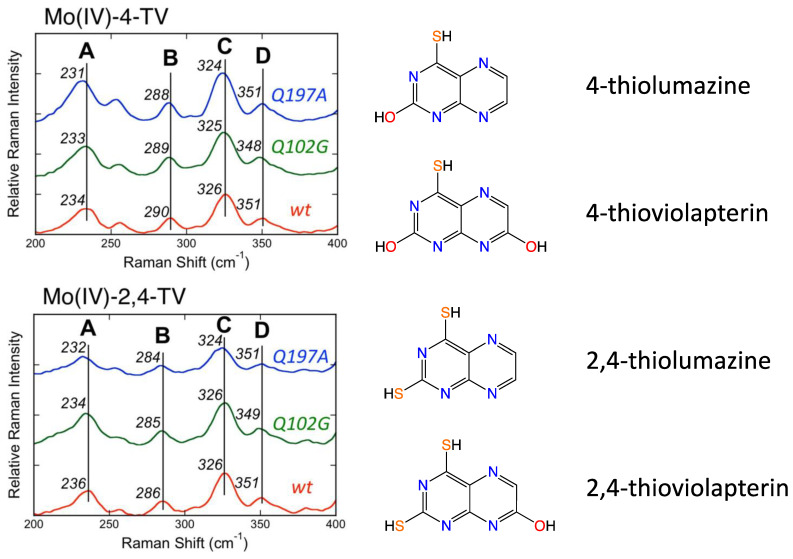
Low-frequency resonance Raman spectra for *wt R. capsulatus* XDH, Q197A and Q102G variants. Adapted with permission from Ref. [90]. Dong, C.; Yang, J.; Reschke, S.; Leimkühler, S.; Kirk, M.L. Vibrational Probes of Molybdenum Cofactor–Protein Interactions in Xanthine Dehydrogenase. *Inorg. Chem*. **2017**, *56*, 6830–6837. https://doi.org/10.1021/acs.inorgchem.7b00028. Copyright 2017 American Chemical Society.

Subsequently, Kirk and coworkers used a combination of electronic absorption and resonance Raman spectroscopies to spectroscopically interrogate the nature of the Mo(IV)-product species in XO/XDH through the use of two different heavy atom congeners of the lumazine substrate (Figure 5) [89,90,97]. The two-electron oxidized 4-thioviolapterin (4-TV) and 2,4-thioviolapterin (2,4-TV) bind tightly to the Mo(IV) centers of wt-XDH and the Q102G and Q197A variants. These important studies provided deep insight into specific Moco-protein interactions. The electronic absorption and rR spectroscopies were evaluated in the context of vibrational and spectroscopic computations, and this enabled an unambiguous assignment of the intense Mo → violapterin charge transfer transition as being a Mo(xy) → violapterin (π*) metal-to-ligand charge transfer (MLCT) excitation [97]. The intensity of this low-energy MLCT band derives from the Mo(xy) redox orbital being oriented orthogonal to the product ring plane, since this allows for strong overlap between the Mo(xy) orbital and the π* orbitals of the thioviolapterin product molecules [89,90,97]. Thus, this MLCT transition can be described as a one-electron promotion from the doubly occupied Mo(xy) orbital to the LUMO of the product, and this effectively produces a hole on the Mo center (e.g., a formal Mo(V) center with the transfer of a full electron). This hole character in the Mo(xy) orbital is generated rapidly with optical excitation and is similar to the hole character generated in the Mo(IV) → Mo(V) electron-transfer process, as electrons are shuttled out of the enzyme to other redox chromophores. Interestingly, the Mo(xy) donor orbital possesses some PDT orbital character that is primarily localized on the in-plane S(p) orbitals of the dithiolene component of the PDT. As a result, the removal of an electron from the Mo(xy) orbital will result in geometric distortions within the entire Mo-dithiolene chelate ring, and these distortions will be similar to those encountered in the electron transfer process. This change in charge distribution following photoexcitation results rR enhancement of key low-frequency Mo-dithiolene ring modes and other low-frequency PDT vibrations that are kinematically coupled to this distortion.

Resonance Raman data for *R. capsulatus* XDH Q102G and Q197A variants display variant-dependent vibrational frequency shifts to lower energy in the low frequency relative to the corresponding spectra of the wt enzyme [90]. The vibrational bands for the Raman spectra in Figure 5 have been assigned, with Band A being described as possessing dithiolene envelope folding character, in addition to both Mo≡O rocking and pyranopterin -NH_2_ twisting character. Low-frequency Mo-dithiolene core vibrational assignments have been assisted by a combination of spectroscopic computations and rR spectra of small-molecule analog compounds that do not possess a pyranopterin moiety fused to the dithiolene chelate as found in the PDT. Band B in Figure 5 is assigned as possessing a combination of Mo-S_dithiolene_, Mo-SH stretching, and pyranopterin -NH_2_ rocking characters, while Band C is assigned as possessing dominant symmetric Mo-dithiolene core stretching characters. Finally, Band D is assigned as a symmetric Mo-dithiolene core stretching vibration with a Mo-SH stretching character. These spectral assignments are highly significant since they provide evidence of either weak hydrogen bonding and/or electrostatic interactions between Q197 and the terminal oxo ligand and between the PDT –NH_2_ terminus and Q102. It was noted that the kinetic parameters k_red_, and k_red_/K_D_ were affected in the Q102G and Q197A variants. This suggests that the primary role of these Glu residues is to assist in the proper positioning of Moco in the active site of XO/XDH. Optical pumping into the Mo(xy) → violapterin (π*) MLCT band results in electron density changes at the Mo site that result in specific geometric distortions in the PDT that extend to the -NH_2_ terminus, and this is reflected in the observed low-frequency Raman shifts between wt XDH and the Q102A variant. The results of this Raman study suggest that there is a functional role for the PDT in electron transfer between the Mo ion and the proximal 2Fe2S cluster for enzymes of the XO family.

### 2.3. What Is Known about Pterin Oxidation State and Pterin Redox Reactivity in Moco

Pterins have been known for more than a half century as small molecules in biology and biochemistry, where they typically function as either pigments or cofactors [103,104,105]. Oxidized pterins are generally found as pigments, while reduced tetrahydropterins participate in enzyme catalysis as redox cofactors. It is therefore logical to consider what possibilities for redox reactivity the reduced pterin component of Moco might employ.

#### 2.3.1. Earliest Redox Studies on PDT in Molybdenum Enzymes

**Figure 6:**
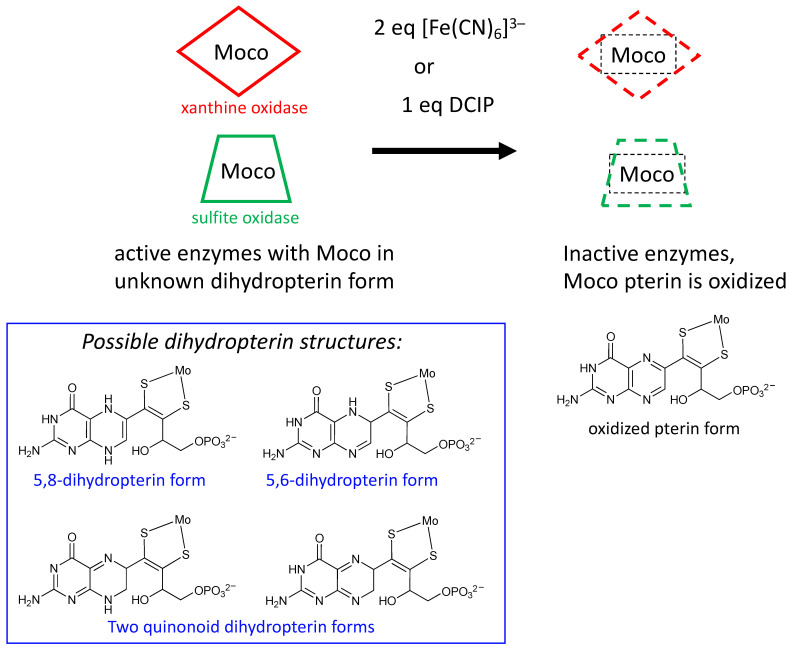
Results from experiments probing the redox state of the pterin in Moco. Enzymes oxidized by either ferrocyanide or DCIP cause inactivation where Moco possesses an oxidized pterin in PDT (top). Oxidation stoichiometry implies active Moco is at the dihydropterin level of reduction having several possible tautomeric structures (bottom).

Rajagopalan was the first to probe the redox state of Moco in several studies initiated shortly after his proposal of its tetrahydropterin structure using detailed absorption spectral analyses [106,107]. The absorption spectrum of XO includes a 300 nm absorption consistent with either a tetrahydropterin or an unstable quinonoid dihydropterin, but it eliminated the possibility of a 7,8-dihydropterin structure. Oxidation of sulfite oxidase and xanthine oxidase by the redox dye dichlorophenylindophenol (DCIP) showed a 2e^−^/2H^+^ reaction occurred at the PDT to produce a fully oxidized pterin that was identified by electronic absorption spectroscopy, and this result indicated that Moco in both XO and SO possesses a pterin at the dihydro-level of reduction [106,107]. On the basis of extensive reactivity studies in the Rajagopalan labs [106,107], the native state of the pterin of Moco in XO was proposed to be a quinonoid dihydropterin, whereas SO was argued to have a different tautomeric dihydropterin structure; suggested possibilities are shown in Figure 6 [106,108]. It was also determined that sulfhydryl groups or sulfides were capable of reducing the putative quinonoid dihydropterin state to the tetrahydro level [106,107]. A later study showed that treating sulfite oxidase with 2 equivalents of ferricyanide abolished catalytic activity as well as the ability of Moco extracts to reconstitute *nit-1* assays [106]. Electronic absorption spectroscopy indicated that ferricyanide oxidized the pterin of Moco to a fully oxidized pterin state that more easily dissociated Mo [106], spurring model studies of the ferricyanide-inhibited sulfite oxidase [109,110]. Rajagopalan concluded from this work that the pterin component of Moco was a dihydropterin of unknown structure and that the pterin was implicated as a participant in catalysis, suggesting possible roles in electron transfer or modulation of the Mo redox potential (Figure 6). These were key studies that drew attention to the nature of pterin reactivity in the Moco, which generated suggestions for how such redox activity might play a role in catalysis as well as for recognizing that the pterin redox level in the PDT can be affected by other prosthetic groups.

#### 2.3.2. Redox Studies on Pyranopterin

**Figure 7:**
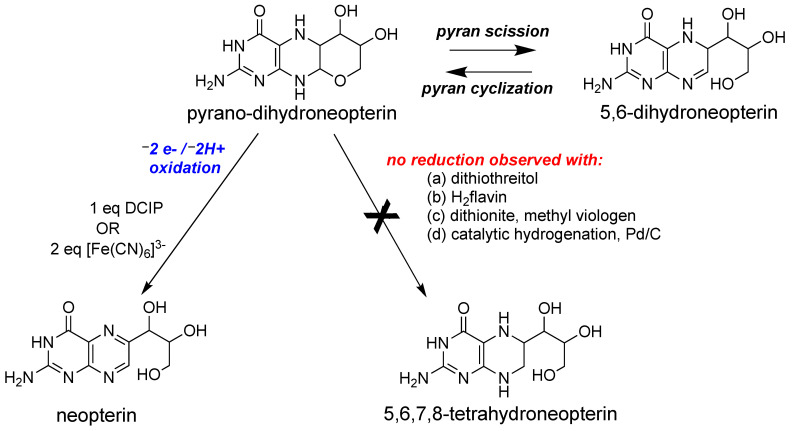
Redox reactivity of a model pyranopterin.

Following the discovery of the pyranopterin structure of PDT [76,77], Burgmayer et al. investigated the redox behavior of a synthetic reduced pyranopterin in reactions with either DCIP or ferricyanide to corroborate the Rajagopalan studies [92,111,112,113]. This work demonstrated that a reduced tetrahydropyranopterin (i.e., the pyrano-dihydroneopterin in Figure 7) reacted as a dihydropterin; that is, it required 1 eq DCIP or 2 eq ferricyanide to generate the oxidized pterin product neopterin (Figure 7). A comparison of the oxidation kinetics of tetrahydropyranopterin vs. 6,7-dimethyltetrahydropterin showed that the reduced pyranopterin was kinetically 400 times slower in oxidation reactions depending on solvent conditions. In contrast to the earlier observations that the pterin in Moco could be reduced to a tetrahydropterin form by sulfide [107], the studies by Burgmayer and coworkers found that reduced pyranopterin failed to undergo further reduction to a 5,6,7,8-tetrahydropterin (Figure 7) when a variety of reductants (dithiothreitol, reduced flavin, dithionite, and Pd/C) were used. The study concluded that reduced dihydropyranopterin has a more robust structure, is slower to oxidize than 5,6,7,8-tetrahydropterin, and is resistant to further reduction. The results highlight the dual personalities of reduced pyranopterin: It behaves as a dihydropterin in oxidations, but it is unreactive under typical biochemical reducing conditions, behaving as though it is fully reduced. These results corroborated the speculations made by Rajagopalan [106,108,114] based on the Moco pterin redox studies performed in his laboratories that Moco possesses a pterin at the dihydropterin level with a structure different from the 7,8-dihydropterin known to be the thermodynamically most stable dihydro tautomer. The pyranopterin structure imparts considerable electronic flexibility to the PDT: structurally, it has a fully saturated pyrazine ring characteristic of a tetrahydropterin, but as mentioned above, it functions as a dihydropterin in redox reactions. These important studies underscore how the frequently used terminology of ‘tetrahydro-pyranopterin’ in PDT research is a misnomer. ‘Tetrahydro’ does convey the nature of the four H atoms in the pyrazine ring but incorrectly addresses the redox chemistry. A more accurate description would be ‘pyrano-dihydropterin’ to designate the structure as the pyran tautomer of the open chain 5,6-dihydropterin.

#### 2.3.3. Role of PDT Oxidation State in Reductive Activation of DMSO Family Enzymes

It is well known that enzymes within the DMSOR family are isolated as heterogeneous samples that can be activated by a prereduction step that reduces unknown species. A recent investigation of such a reductive activation in nitrate reductase concludes with the proposal that it is the pterin of PDT that requires reduction [115,116,117]. Dissimilatory *E. coli* nitrate reductase (Ec Nar) was studied using protein film voltammetry to obtain kinetic parameters for the reductive activation [115]. Based on the kinetic analysis, there are two inactive species in equilibrium in the Nar enzyme, and only one of these is reductively activated by sodium dithionite. Furthermore, it is proposed that the equilibrium involves the cyclization of an open pterin form of PDT to a cyclized pyranopterin form of PDT prior to the reduction step that produces the active Nar enzyme. An explanation of the special role of pyranopterin in accessing a necessary reduction step is provided by results from synthetic Moco models as described in Section 3.2.

#### 2.3.4. Pterin Protein Environment in DMSOR Family Enzymes Correlates with Mo Reduction Potential

Nitrate reductase NarGHI from *E*. *coli* was the first enzyme identified by protein crystallography to possess a Moco structure with one pyranopterin (proximal) and one bicyclic, pyran-opened pterin (distal) (Figure 2d) [81]. Variants were made at amino acid residues having H-bonding interactions at the O atom of the open, distal PDT pterin (Figure 2d) to assess the effect on the Mo redox potential E_m_ [118]. It was found that when Ser719 was replaced by alanine, there was very little effect on Mo E_m_, whereas the H1163A and H1184A variants caused large effects (ΔE_m_ values of −88 and −36 mV, respectively). On this basis, it was proposed that a charge transfer relay involving both His residues and three water molecules regulates the protonation state of the pyran-OH and thereby the Mo reduction potential. This charge relay was also proposed as initiating the pyranopterin ring opening reaction of the distal PDT via proton abstraction. A second mutation investigated the amino acid bridging the proximal and distal pterins at their N5 atom positions within each pterin. For NarGHI nitrate reductase and most members of the DMSOR family, this bridging residue is a histidine (His1092 in Figure 4d), whose H-bonding to the proximal PDT at pterin N5 is believed to maintain the reduced pyranopterin structure. Alanine variants of His1092 and His1098 also caused large ΔE_m_ values of −143 and −101 mV, respectively. The results of this study support the hypothesis that changes in the pterin component of the PDT, both in terms of its oxidation state and its structure (or tautomeric form), can affect the Mo reduction potential. This modulation of the reduction potential may be used to tune an enzyme to function with a variety of substrates, thereby explaining the diversity of substrate transformations performed by enzymes in the DMSOR family.

A similar outcome was obtained from a study directed at destabilizing factors for the Mo(V) state in arsenite oxidase [119]. Here, Arg720 serves as a bridge between the proximal and distal PDT ligands. However, it is Gln726 that H-bonds through an amide O atom to the proximal pyranopterin N5 site, which plays an important role in stabilizing the reduced pyranopterin. Mutating glutamine to glycine altered Mo reduction potentials and the stability of the Mo(V) state, from which the study concludes that the H-bond environment of the pyranopterins controls the potential of each Mo redox step, where elimination of the H-bonding interaction ultimately stabilized the intermediate Mo(V) state and dramatically changed a cooperative 2e^−^ process to a two 1e^−^ step process.

## 3. What Has Been Learned about Moco from Model Studies Directly Probing PDT-Mo Interactions?

Duplicating key geometric and electronic structural features of Moco through the synthesis of biomimetic model compounds has long been a strategy employed by inorganic chemists who seek to develop a more comprehensive understanding of how the molybdenum cofactor contributes to catalysis. Now that we know the structural components of the special PDT ligand that binds Mo to form the active cofactor, it may seem that model studies are no longer needed or useful for providing further information on this complex cofactor. One needs only to return to the several features highlighted in the introductory section of this review to identify critical areas where detailed examination of model compounds can provide information that cannot be easily extracted from studies on the protein-bound cofactor. For this reason, we have devoted considerable effort toward investigations geared toward understanding the chimeric dithiolene chelate and the subtleties of how a pyranopterin further modifies the dithiolene moiety coordinated to molybdenum to create what is arguably the most non-innocent ligand to be found in biology. The following sections highlight recent accomplishments of this work. Since the focus of this review is limited to studies of pyranopterin dithiolene model complexes of molybdenum and how these results relate to the enzyme systems, reports of synthetic pterins and pyranopterins lacking a dithiolene coordinated to molybdenum are outside the scope of the review [120,121].

### 3.1. Studies That Define “Simple” Mo-Ditholene Interactions

#### 3.1.1. Tp*MoO(bdt)

**Figure 8:**
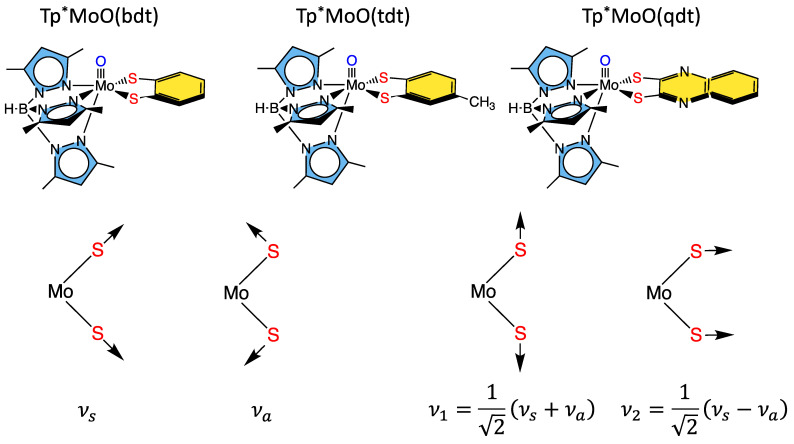
Top: Tp*MoO(dithiolene) first-generation model complexes that have been extensively probed spectroscopically using a combination of MCD, electronic absorption, photoelectron, electron paramagnetic resonance, and resonance Raman spectroscopies. Bottom: Symmetry coordinates for two totally symmetric low-frequency normal modes and their respective linear combinations.

Some of the first comprehensive spectroscopic studies on oxo-molybdenum dithiolene model complexes were performed by Kirk and Enemark on Tp*MoO(dithiolene) complexes (Tp* = *tris*-(3,5-dimethylpyrazolyl)hydroborate; dithiolene = bdt, tdt, qdt) (Figure 8) [2,84,91,122]. This work showed evidence for low-energy dithiolene → Mo LMCT transitions that indicated a three-center, pseudo-σ, Mo(xy)—S(dithiolene) bonding interaction is present in this system. From an electron transfer viewpoint, these results supported the hypothesis that in-plane Mo-S covalency could be important in modulating active site reduction potentials by destabilizing the Mo(xy) redox orbital in mono-oxo sites. For mono-oxo enzyme active sites [84,123,124], the strong ligand field produced by the Mo≡O bond orients the Mo(xy) orbital to be orthogonal to this bond, with implications for both atom and electron transfer reactivity [123]. Thus, if the pyranopterin component of the PDT is involved in electron transfer regeneration of catalytically competent active sites, there must be a long-range superexchange pathway that couples the Mo(xy) redox orbital into the PDT [84,110,125]. The low-frequency rR spectra of these key molecules show two important totally symmetric vibrational modes that can be described: S-Mo-S stretching and bending (Figure 8, bottom left) [2,84,91,122]. Since these vibrations have the same symmetry, they can mix to yield the vibrational modes given at the bottom right of Figure 8. These vibrations, in addition to the Mo-dithiolene S-S folding distortion, can dynamically affect the degree of Mo-S_dithiolene_ covalency, and these geometric distortions may conspire to vibronically couple the enzyme active site into hole superexchange pathways for one-electron transfer reactivity in the electron transfer half reaction.

#### 3.1.2. Remote Charge Effects on Oxygen Atom Transfer Reactivity

Differences in the electron-donating ability of the PDT, which could result from S-fold distortions, contributing thiol-thione resonance forms, PDT protonation, etc., possess the potential to affect the rates of oxygen atom transfer reactivity in pyranopterin Mo enzymes. Recent model compound studies have shown that changing the molecular charge by a single unit at a position remote from the Mo ion can have dramatic effects on thermodynamic parameters and reaction kinetics related to oxygen atom transfer reactivity [126]. In comparing Tp*Mo^VI^O_2_Cl with [Tpm*Mo^VI^O_2_Cl]^1+^, differences in their respective molecular charges arise from a single atom substitution (N → C). The change in charge at the virtual parity of their geometric structures leads to a dramatic +350 mV change in the Mo^VI^/Mo^V^ reduction potential. A comparative analysis of the frontier molecular orbitals and electrostatic potential energy surfaces between Tp*Mo^IV^O_2_Cl and [Tpm*Mo^IV^O_2_Cl]^1+^ showed that the remarkable shift in the reduction potential can be explained by a stabilization of the [Tpm*Mo^IV^O_2_Cl]^1+^ LUMO. This LUMO stabilization results in an increase in the oxygen atom transfer reaction rate by several orders of magnitude, and the observed rate acceleration was accompanied by a larger thermodynamic driving force in accordance with the Bell-Evans-Polanyi principle. Thus, the Mo reduction potential in the enzymes can be modified by a few hundred mVs with changes in charge that are remote from the Mo center. This charge effect study conclusively showed that the structural changes that accompany charge changes are likely to be difficult or even impossible to observe in the enzymes using protein X-ray crystallography.

#### 3.1.3. Mo-Dithione Interactions Relevant to Molybdoenzymes

Although the Mo ion redox cycles between the Mo(IV) and Mo(VI) states in most molybdoenzymes, with one-electron nitrite to NO• and the tungstoenzyme-catalyzed non-redox hydration of acetylene being notable examples [28,29,30,31,32,127,128,129,130], the PDT has not been shown to be redox active in catalysis [48], although it is capable of storing up to six redox equivalents. Two of these equivalents are localized on the dithiolene, and four are localized on the pterin. Spectroscopic and electronic structure studies on [Mo^4+^O(*i*Pr_2_Pipdt)_2_Cl][PF_6_] (Pipdt = *N*,*N*-piperazine-2,3-dithione) have been used to explore the potential non-innocence of the dithiolene in PDT [131]. The electronic absorption spectrum of this complex is unusual for a Mo(IV) complex in that it possesses a relatively intense (ε ~ 1400 M^−1^cm^−1^) low-energy (E ~ 13,500 cm^−1^) metal-to-ligand charge-transfer (LMCT) band. Typically, low-energy LMCT transitions in mono-oxo Mo sites are not observed due to the large terminal oxo-derived ligand field splitting of the t_2g_ orbitals and the double occupancy of the lowest energy Mo(xy) orbital. However, if the dithiolene is oxidized to a dithione and ligand acceptor orbitals are available, low-energy MLCT may be observed. This is the case for [Mo^4+^O(*i*Pr_2_Pipdt)_2_Cl]^1+^, where the MLCT has been assigned as Mo(xy) → dithione(π*) HOMO → LUMO transition based on spectral computations and resonance Raman enhancement of bands with C–C and C–S stretching characters. The *i*Pr_2_Pipdt ligand was described in valence bond terms using a natural bond orbital approach to be comprised of a hybrid of contributing dithione (63%) and di-zwitterionic dithiolene (37%) resonance structures. The π-acceptor character of this type of dithione was also shown in studies on MoO(SPh)_2_(*i*Pr_2_Dt_0_) (*i*Pr_2_Dt_0_ = N,N′-isopropyl-piperazine-2,3-dithione), where an intense thiolate → dithione ligand-to-ligand CT band was assigned at ~18,000 cm^−1^. This assignment was based on spectroscopic computations and resonance Raman enhancement of a 378 cm^−1^ vibration that was shown to possess dithione ligand S−Mo−S + C−N stretch character. The π-acceptor character of the ligand is also exemplified in a dramatic dithione ligand fold angle distortion of 70°, which derives from the pseudo-Jahn–Teller effect [91]. Here, excited state—ground state mixing is mediated by a low-frequency vibration that drives the large fold-angle distortion. This results in a warped, double-well, ground-state potential energy surface. However, we know of no evidence in any enzyme crystal structure where such a dramatic S-S ligand fold distortion is present, and thus the existence of a dominant π-acceptor dithione form of the PDT dithiolene in an enzyme has yet to be confirmed.

#### 3.1.4. Donor-Acceptor Quinoxaline Dithiolene Ligands

**Figure 9:**
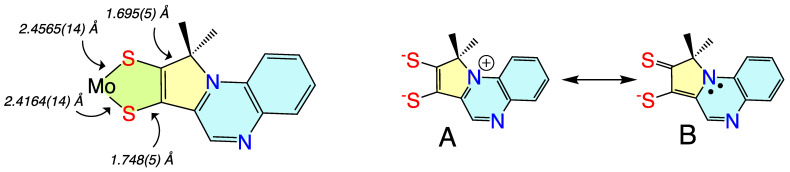
(left) Bond lengths determined from X-ray crystallography for Tp*MoO(pyrrolo-S2BMOQO). (right) Contributing resonance structures for the ligand showing dominant dithiolene (**A**) and thione-thiolate (**B**) structures.

Non-innocent metal-ligand redox behavior in molybdenum dithiolene complexes that possess ligands comprised of nitrogen heterocycles was initially reported by Pilato in a series of pyridinyl- and quinoxalinyl- dithiolene complexes of molybdenum of the type Cp_2_Mo(S_2_C_2_(heterocycle)H) [132], foreshadowing the results we would obtain using pyranopterin dithiolene ligands. The potential for such non-innocent behavior in the molybdenum cofactor was originally demonstrated using a ligand (pyrrolo-S_2_BMOQO) comprised of an N-heterocycle (quinoxaline) that is appended to a dithiolene fragment that was covalently bound to a Mo(IV) ion [133]. These quinoxalyldithiolene ligands effectively served as first-generation models for how the PDT may function in Moco. Tp*MoO(pyrrolo-S_2_BMOQO) is formed from the dehydration of TEA[Tp*MoO(S_2_BMOQO)] (TEA = tetraethylammonium; Tp* = hydrotris(3,5-dimethylpyrazolyl)-borate), where an intramolecular cyclization within the S_2_BMOQO ligand occurs. A combination of DFT computations, which were interpreted in the context of resonance Raman and electronic absorption spectroscopies and complemented by X-ray crystallographic studies, revealed that an asymmetric dithiolene chelate was present in Tp*MoO(pyrrolo-S_2_BMOQO). Additionally, it was shown that this five-membered MoS_2_C_2_ chelate ring possessed considerable thione-thiolate character. A valence bond description was used to describe the observed Mo-ligand chelate ring thione-thiolate bonding character, and this analysis showed that there were two dominant resonance structures that contribute to the electronic structure description (Figure 9). One of the resonance structures is that of a symmetric dithiolene, while the second is an asymmetric resonance structure that derives from a redistribution of electrons between the dithiolene and quinoxaline components of the pyrrolo-S_2_BMOQO ligand. A significant contribution from the thione-thiolate resonance structure conveniently explains the chelate ring asymmetry found in the crystal structure of the complex. This electronic structure description of the ligand also explains the appearance of a dithiolene → quinoxaline intraligand CT band in the electronic absorption spectrum. Additionally, the pyrrolo-S_2_BMOQO ligand is also highly electron-withdrawing, and this further highlights the potential non-innocent behavior of this and similar dithiolene ligands. This reveals a potential key role for the pterin N-heterocycle to function similarly in the enzymes by modulating the electron-donating ability of the dithiolene sulfur donors to control substrate transformation and electron transfer redox processes at the Mo ion during catalysis. This critical observation and electronic structure description of a thione-thiolate type ligand was later observed in oxo-Mo(IV) dihydropterin complexes [92].

### 3.2. Model Systems That Incorporate Both Dithiolene and Pyranopterin Structures on Molybdenum

The first Moco model compound to successfully incorporate a pterin dithiolene ligand on a Mo(4+) ion was reported from Pilato’s labs in 1991 [134,135]. These Cp_2_Mo(IV)(S_2_C_2_(pterin)(COMe)) systems were constructed on a *bis*-cyclopentadienyl-Mo(IV) structure that lacked a terminal oxo ligand. Limited studies of this molecule demonstrated one electron oxidation to Mo(V) and reactivity towards acids. Subsequently, Garner and coworkers [136] reported a pterin dithiolene ligand in a related complex, CpCo(S_2_C_2_(pterin)(H)).

**Figure 10:**
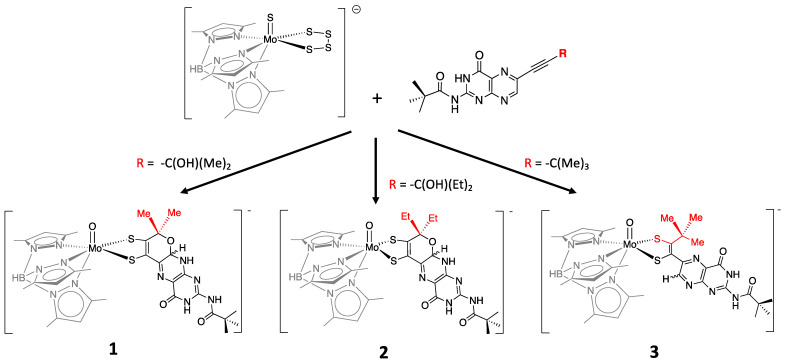
Pterin-dithiolene model compounds for Moco.

More recently, a number of pterin- and quinoxaline-dithiolene Mo compounds have been designed in the Burgmayer labs. Each is synthesized from the reaction of a molybdenum tetrasulfide precursor [Tp*MoS(S_4_)]^−^ with a suitably substituted pterinyl- or quinoxalyl-alkyne, as depicted in Figure 10. The pivaloyl group added to the exocyclic amine group of the pterin overcomes the notorious insolubility of pterins. Those complexes shown in Figure 10 have been studied in detail to provide considerable insight about the (pterin-dithiolene)-Mo system.

#### 3.2.1. Pyranopterin Impact on Dithiolene

**Figure 11:**
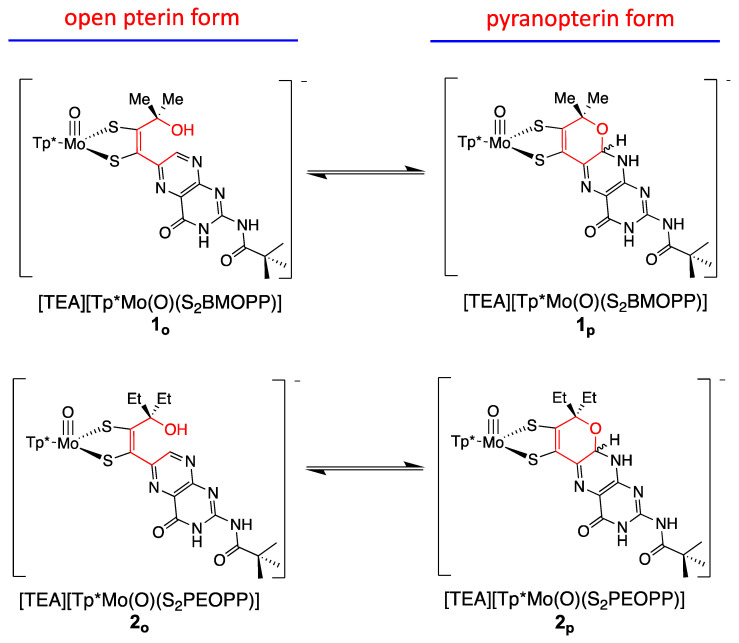
Model compounds [Tp*MoO(S_2_BMOPP)]^1−^ (**1**) and [Tp*MoO(S_2_PEOPP)]^1−^ (**2**) spontaneously cyclize, forming a pyran ring yielding the pyranopterin dithiolene structure found in Moco, and demonstrate pyran ring-chain tautomerization. In contrast, model compound [Tp*MoO(S_2_BDMPP)]^1−^ (**3**) cannot form a pyran ring.

The three model compounds in Figure 10 were investigated in a suite of experiments to determine the effect of the pterin redox state on the dithiolene chelate and, in turn, on the Mo atom [111]. Both complexes **1** and **2** were observed to exist as an equilibrium mixture of two pterin forms: the pyran ring cyclized and the ring opened (Figure 11). The equilibrium between open and cyclized forms was discovered to be sensitive to the solvent environment, where more polar solvents (ACN, DMSO) stabilize the pyranopterin form while non-polar solvents (CHCl_3_, THF) favor the ring-opened form. The difference in pterin conformation leads to a difference in pterin orientation with respect to the dithiolene, which in turn causes a modest but significant difference in the electronic environment of the Mo atom that can be quantified by electrochemistry, electronic absorption, and infrared spectroscopies. However, because the open form **1_o_** cannot be obtained without the presence of the pyrano form **1_p_**, complex **3** (Figure 10) was designed to allow measurements on an open pterin model complex that cannot undergo cyclization. The Mo atom in **1_p_** is relatively electron deficient, as indicated by a Mo(V/IV) reduction potential shift of ~+54 mV for the pyrano form **1_p_** as compared to the open form **3** and, by analogy, **1_o_**. This is consistent with the higher Mo≡O stretching frequency of **1_p_** (+8 cm^−1^) compared to **3**. The reason for this electronic change felt at Mo is fundamentally due to the pyran ring presence, which constrains the pterin ring system to be nearly planar to the dithiolene chelate. This co-planar pterin-dithiolene arrangement allows for conjugation to be extended from the Mo atom through the entire pterin ring system.

#### 3.2.2. The Electronic Origin of the Thione-Thiol Resonance Form and Implications for Pyranopterin-Mo Enzymes

We have used electronic absorption spectroscopy to probe the electronic structure differences between the pyran ring-closed [Tp*MoO(S_2_BMOPP)]^1−^ (**1**) and the ring-opened compound [Tp*MoO(S_2_BDMPP)]^1−^ (**3**) [111]. The primary goal of this work was to understand the electronic origin of the +54 mV shift in their respective reduction potentials as a function of extended delocalization between Mo and the pterin ring system. This electron delocalization in the ligand is modulated by pyran ring opening and closing, leading to distinct spectroscopic signatures for these complexes. Specifically, the electronic absorption spectrum of pyran ring-closed [Tp*MoO(S_2_BMOPP)]^1−^ possesses lower energy charge transfer bands of greater intensity than those observed for [Tp*MoO(S_2_BDMPP)]^1−^, and the electronic absorption spectrum of [Tp*MoO(S_2_BDMPP)]^1−^ is markedly broader compared to that of [Tp*MoO(S_2_BMOPP)]^1−^. The broadened spectral features most likely reflect the presence of a variety of rotomeric contributions to the spectrum that result from distortions about the dithiolene-pterin C-C bond.

**Figure 12:**
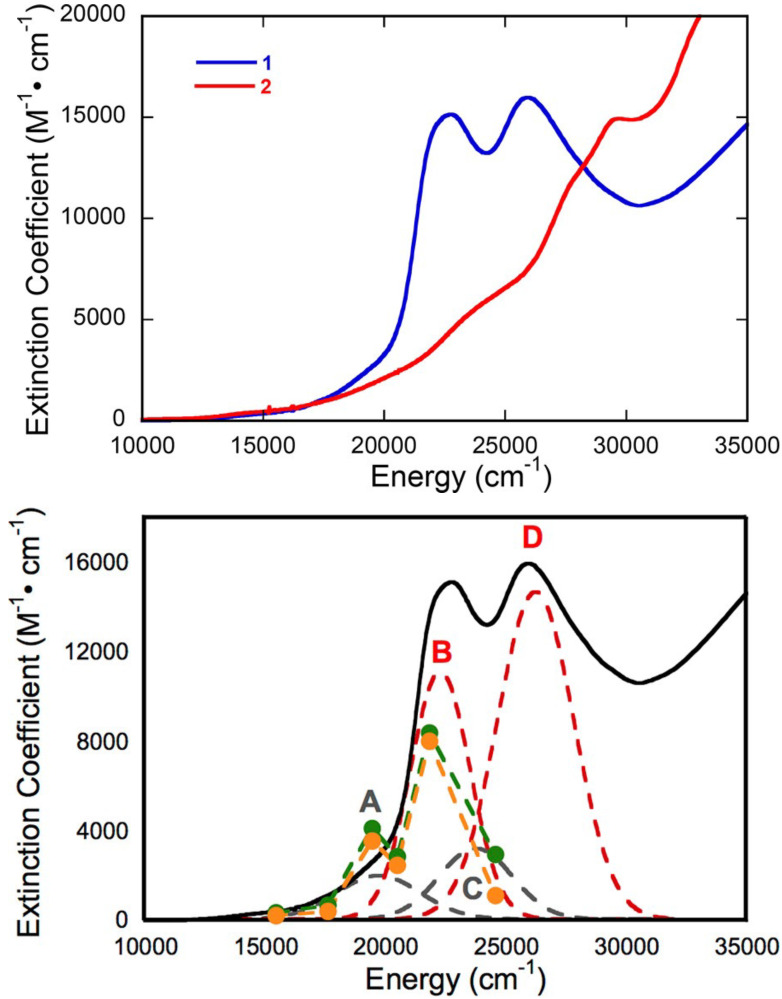
Electronic absorption spectra (298 K in DMSO) of [Tp*MoO(S_2_BMOPP)]^1−^ (1) and ring-opened [Tp*MoO(S_2_BDMPP)]^1−^. Bands A–D derive from a Gaussian resolution of the electronic absorption spectrum. Adapted with permission from Ref. [111]. Gisewhite, D.R.; Yang, J.; Williams, B.R.; Esmail, A.; Stein, B.; Kirk, M.L.; Burgmayer, S.J.N. Implications of Pyran Cyclization and Pterin Conformation on Oxidized Forms of the Molybdenum Cofactor. *J. Am. Chem. Soc.* **2018**, *140*, 12808–12818. https://doi.org/10.1021/jacs.8b05777. Copyright 2018 American Chemical Society.

The electronic absorption spectrum of [Tp*MoO(S_2_BMOPP)]^1−^ has been assigned with the help of resonance Raman excitation profiles and spectroscopic computations at the DFT level of theory (Figure 12) [111]. Here, Band A derives from a combination of both Mo d(xy) → pterin (HOMO → LUMO) charge transfer and Mo d(xy) → Mo(xz) ligand field one-electron promotions, while Band B can be described as an intraligand charge transfer (ILCT) transition that is dominated by a dithiolene → pterin one-electron promotion. Further support for the charge transfer nature of these two bands and the direct involvement of the dithiolene moiety in these transitions comes from resonance Raman spectroscopy, where excitation into both bands A and B both result in resonance enhancement of two high-frequency vibrations with dithiolene C=C stretching character. Band C was assigned as a LMCT transition and Band D as another dithiolene → pterin ILCT transition that derives from a HOMO-2 → LUMO one-electron promotion. Thus, the partially oxidized pterin system is observed to be electron withdrawing with respect to the dithiolene, leading to intense intraligand charge transfer transitions that are signatures of a conjugated pterin-dithiolene ligand. However, when the pyran ring of the pterin dithiolene ligand is open, steric constraints force the pterin ring to rotate out of the dithiolene plane. This rotation is important because it results in a loss of conjugation. Spectroscopically, this is observed as an absence of intense ILCT charge transfer transitions in the electronic absorption spectrum [137].

**Figure 13:**
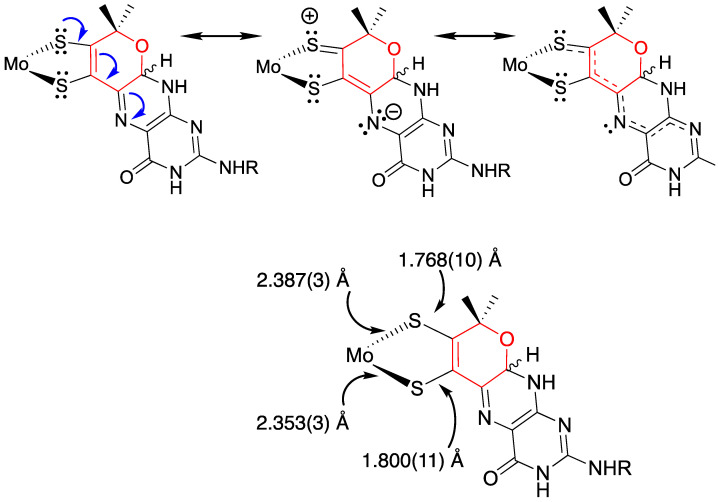
Contributing resonance structures leading to π delocalization in Moco, as evidenced by the bond alternation patterns in the dithiolene chelate ring. Adapted with permission from Ref. [111]. Gisewhite, D.R.; Yang, J.; Williams, B.R.; Esmail, A.; Stein, B.; Kirk, M.L.; Burgmayer, S.J.N. Im-plications of Pyran Cyclization and Pterin Conformation on Oxidized Forms of the Molybdenum Cofactor. *J. Am. Chem. Soc.* **2018**, *140*, 12808–12818. https://doi.org/10.1021/jacs.8b05777. Copyright 2018 American Chemical Society.

Understanding the nature of these spectra is important because they reveal the key spectroscopic signatures of ring-closed and partially oxidized PDTs, in addition to ring-opened PDTs. Critically, the key spectroscopic signature for the presence of a PDT containing a dihydropyranopterin with a closed pyran ring is the aforementioned intense dithiolene → pterin CT transition in the visible region of the electronic absorption spectrum. These ILCT transitions also reflect the push-pull donor-acceptor nature of partially oxidized PDTs and provide additional support for the configurational mixing of the ILCT excited state into the electronic ground state, resulting in the observed dithiolene chelate ring bond asymmetry we find in the X-ray structure of [Tp*MoO(S_2_BMOPP)]^1−^ (Figure 13). It is important to note that the resonance structures shown in Figure 13 reflect how the dithiolene → pterin ILCT character can be admixed into the electronic ground state. This resonance structure description also illuminates the reason why this type of dithiolene ligand is a poorer electron donor to the Mo ion, contributing to the +54 mV shift in the reduction potential of [Tp*MoO(S_2_BMOPP)]^1−^ relative to the ring-opened [Tp*MoO(S_2_BDMPP)]^1−^ complex. S K-edge XAS has also been employed to gain insight into the relative electron-donating ability of the dithiolene sulfurs in [Tp*MoO(S_2_BMOPP)]^1−^ and [Tp*MoO(S_2_BDMPP)]^1−^, and the results of these experiments also support our assertion that the dithiolene in [Tp*MoO(S_2_BMOPP)]^1−^ is the poorer electron donor to the Mo ion [111]. These data also support the existence of low-energy virtual pterin-based ligand orbitals with a low degree of S orbital character, which is consistent with our observation of low-energy dithiolene → LUMO (pterin) ILCT transitions in the electronic absorption spectrum of [Tp*MoO(S_2_BMOPP)]^1−^.

The results of these studies show that the pyranopterin moiety can be electronically coupled into the Mo-dithiolene fragment when the pyran ring is intact (ring-closed) and both the dithiolene chelate ring and the pterin rings of the PDT are in a relatively coplanar arrangement. As a result, electron-deficient pterins are capable of stabilizing electron-rich Mo(IV) ions via a redistribution of charge in order to affect catalytic transformations and electron transfer reactivity. In summary, the studies outlined here indicate that reversible pyran cyclization and scission may function as switches to enable dynamic electronic coupling/decoupling of the dithiolene and pterin units during catalysis. The overall effect would be to modulate or control the active site reduction potential of the enzymes.

#### 3.2.3. Implications of Pyran Cyclization on PDT Oxidation State and Pterin Conformation in Moco

**Figure 14:**
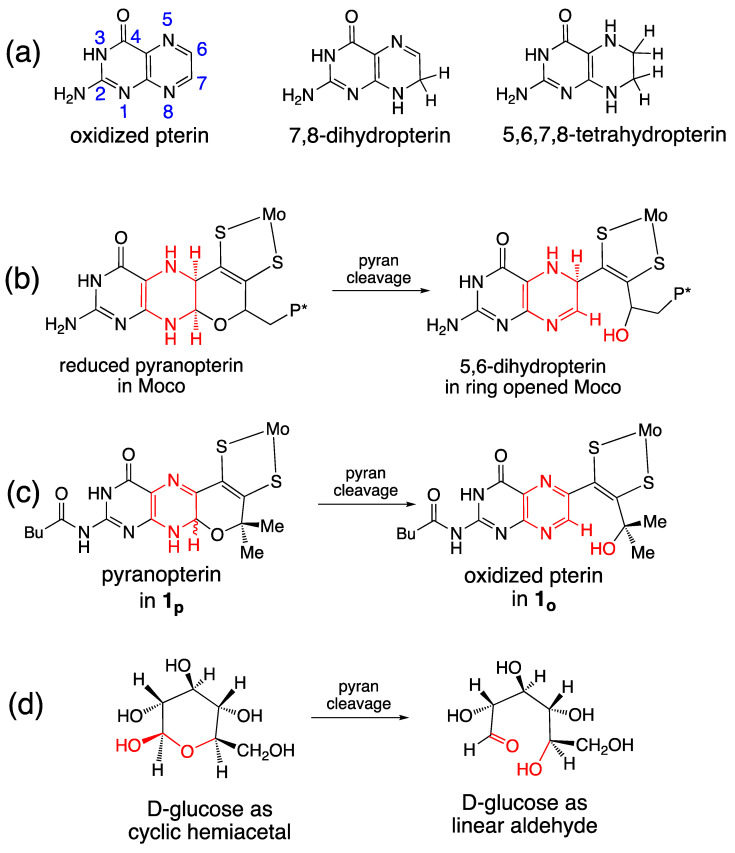
Pterin oxidation states and pyran ring opening outcomes for Moco and model compound **1**. (**a**) Three oxidation levels of pterins. (**b**) Pyran ring opening in Moco. (**c**) Pyran ring opening in **1**. (**d**) Ring-chain tautomerism in D-glucose.

Our studies of the reversible pyran ring cleavage and cyclization observed in model complexes have revealed that pyranopterin has the unusual ability to access pterin forms in effectively different oxidation states without a net loss or addition of electrons [92]. This is illustrated in Figure 14a, which shows the three oxidation levels of a simple pterin, and Figure 14b,c which shows two examples where pyran ring cleavage produces a pterin structure that is electronically equivalent to a 2e^−^/2H^+^ pterin oxidation, yet no *net redox* reaction occurred. In Figure 14b, the pyran ring opening in the reduced pyranopterin form of Moco reveals a partially oxidized dihydropterin structure that is electronically equivalent to the pyranopterin form of **1_p_**. In Figure 14c, the pyran ring opening of **1_p_** generates the fully oxidized pterin structure. Note that these oxidation state assignments also reflect the redox titration studies described earlier in Section 2.3.2. Hence, the pyranopterin component of the PDT can shuttle between oxidized or reduced states without a net loss/addition of electrons. This process mimics the ring-chain tautomerism [138] of linear aldehyde and cyclic hemiacetal structures in carbohydrates, shown in Figure 14d. Viewed within the frame of a ring-chain tautomerism, the PDT of Moco is correctly described as a dihydropterin that is in a protected, cyclic hemiaminal form.

Based on our detailed analyses of the electronic structures of model compounds having open- and pyrano-pterin structures (Section 3.2.2) [111], we expect that when the PDT is ring-closed or semi-oxidized, there will be thione-thiolate [92,139] resonance contributions to the dithiolene chelate that increase dithiolene chelate ring asymmetry. The consequence will be a modification of Mo-PDT electron delocalization.

The reductive activation required by certain enzymes, presented in Section 2.3.3, can now be understood based on the results obtained for the above model compounds **1** and **2**. The two conformers of model **1**, i.e., the open and pyranopterin forms (Figure 11), exist in equilibrium with a K_eq_ value close to that determined for the Nar enzymes [115] that require reductive activation (Section 2.3.3), thereby confirming the proposed pre-equilibrium required for reductive activation. The sensitivity of the dithiolene electronic structure in the presence of a pyran ring provides the basis for understanding the more positive reduction potential of ring-closed PDTs compared to their ring-opened form and yields an explanation of why the pyrano-form of the pterin-dithiolene ligand must be formed prior to reduction.

### 3.3. Modeling Pterin Protonation in PDT Reveals the Indivisible Mo-Pterin-Dithiolene System

The PDT ligand of Moco is rich with functional groups, implicating an important role for hydrogen bonding at the active site. Protein crystal structures have indeed shown a plethora of H-bonds tethering Moco to the protein through the PDT ligand, and this suggests that one role of the pterin is to anchor the cofactor in the proper orientation for catalysis. One can speculate whether such H-bonding interactions might also be used to tune reactivity. To address this question, this section describes a study of outcomes from pterin protonation using model systems **1** and **3** treated with trifluoroacetic acid (TFAA). The aim of this study was to model how proton transfer from neighboring acidic amino acid residues in the protein PDT binding pocket might impact the electronic structure of the Mo-PDT unit in Moco. This is important since several earlier studies demonstrated that protonation of simple N-heterocycles on a dithiolene ligand shifts the redox potential at the metal [132,140,141].

#### 3.3.1. Pyranopterin Structure Enhances Pterin Protonation

**Figure 15:**
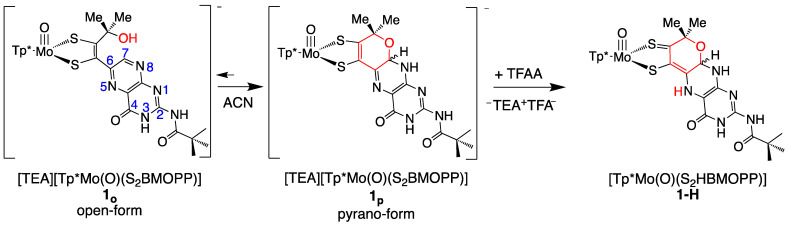
Reaction of model compound **1** with trifluoroacetic acid. The dithiolene chelate is drawn as a partially oxidized thione/thiolate group based on experimental and computational data on the electronic structure of **1-H**. Reproduced with permission from Ref. [92]. Gates, C.; Varnum, H.; Getty, C.; Loui, N.; Chen, J.; Kirk, M.L.; Yang, J.; Nieter Burgmayer, S.J. Protonation and Non-Innocent Ligand Behavior in Pyranopterin Dithiolene Molybdenum Com-plexes. *Inorg. Chem*. **2022**, *61*, 13728–13742. https://doi.org/10.1021/acs.inorgchem.2c01234. Copyright 2022 American Chemical Society.

Protonation of model compounds **1** and **3** was investigated in acetonitrile (ACN), where **1** is >90% in the cyclized pyranopterin conformation [92]. Since **3** lacks an -OH group, it adopts only the uncyclized, open structure where the pterin is rotated ~40° away from the dithiolene plane. Based on observed changes in NMR, electrochemistry (cyclic voltammetry), and UV/vis measurements, **1** is protonated by the addition of 1–2 eq TFAA forming **1-H**, while 3 shows minimal protonation even on the addition of excess acid. Figure 15 shows that protonation of **1** occurs at pyranopterin position N5. The basicity of the N5 position can be understood as a direct outcome of the thione/thiolate resonance structure [86], which increases electron density at N5 through delocalization enhanced by the coplanarity of pyranopterin and dithiolene. Surprisingly, **1-H** exists exclusively in the pyranopterin form, as observed by ^1^H NMR. Therefore, protonation at the pterin N5 position stabilizes the pyranopterin structure and completely disfavors pyran ring opening.

**Figure 16:**
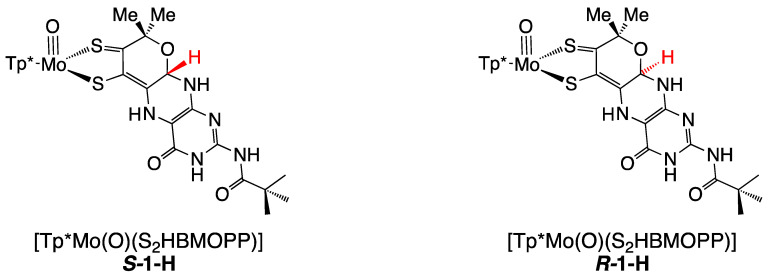

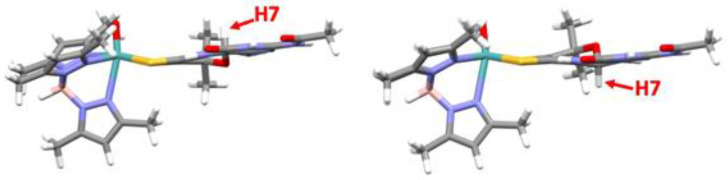
DFT-optimized structures for R- and S-diastereomers of **1-H**. Torsion angles between calculated dithiolene plane (atoms S-C=C-S) and pterin plane are 4.85 degrees for S-H7 and 2.80 degrees for R-H7. Reproduced with permission from Ref. [92]. Gates, C.; Varnum, H.; Getty, C.; Loui, N.; Chen, J.; Kirk, M.L.; Yang, J.; Nieter Burgmayer, S.J. Protonation and Non-Innocent Ligand Behavior in Pyranopterin Dithiolene Molybdenum Com-plexes. *Inorg. Chem*. **2022**, *61*, 13728–13742. https://doi.org/10.1021/acs.inorgchem.2c01234. Copyright 2022 American Chemical Society.

The protonated model **1-H** is highly reactive to oxidants and has limited stability, which has prevented molecular structure determination by crystallography. Instead, DFT optimizations were used to confirm N5 as the favored protonation site and to obtain bond lengths and the pterin conformation. Pterin protonation increases the co-planarity of the pterin and dithiolene portions of the ligand, where optimized *R*- and *S*- diastereomers exhibit slightly different conformations (Figure 16).

#### 3.3.2. Pterin Protonation Strongly Affects the Electronic Structure of Mo-Dithiolene

**Figure 17:**
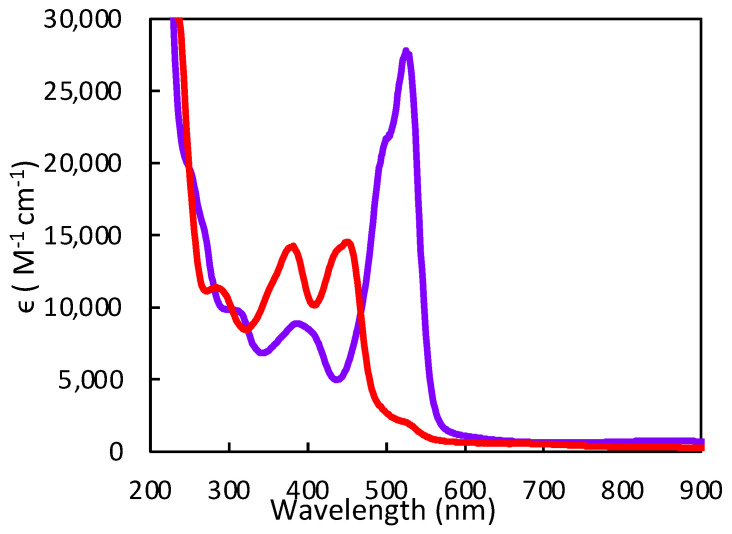
Room-temperature electronic absorption spectra of **1** (red) and **1-H** (purple) in ACN. Adapted with permission from Ref. [92]. Gates, C.; Varnum, H.; Getty, C.; Loui, N.; Chen, J.; Kirk, M.L.; Yang, J.; Nieter Burgmayer, S.J. Protonation and Non-Innocent Ligand Behavior in Pyranopterin Dithiolene Molybdenum Com-plexes. *Inorg. Chem*. **2022**, *61*, 13728–13742. https://doi.org/10.1021/acs.inorgchem.2c01234. Copyright 2022 American Chemical Society.

Protonation of [Tp*MoO(S_2_BMOPP)]^1−^ results in dramatic changes in the electronic absorption spectrum, *vide supra* (Figure 17). Upon addition of TFAA, [Tp*MoO(S_2_BMOPP)]^1−^ is protonated at the N5 position, where the electronic absorption spectrum is dominated by an intense band at 19,000 cm^−1^ (ε ∼ 27,500 M^−1^ cm^−1^) that is assigned as arising from two nearly isoenergetic transitions that can be described as linear combinations of HOMO-1 → LUMO and HOMO → LUMO one-electron promotions with dithiolene → pyranopterin ILCT character and Mo(xy) → pyranopterin MLCT character. Protonation of [Tp*MoO(S_2_BMOPP)]^1−^ dramatically changes the electronic structure of the complex by increasing the degree of the thione-thiolate resonance structure contribution, which is known to increase the dithiolene chelate bond asymmetry. This bond asymmetry (e.g., the % thione character in the chelate) was found to correlate linearly with changes in the chelate C-S bond distance [92]. Furthermore, only the thiolate S of the thione-thiolate contributes to the dithiolene → pyranopterin ILCT, resulting in a dithiolene ligand that is a poorer electron donor than that found in the non-protonated form. Remarkably, protonation at N-5 increases the Mo(V)/Mo(IV) reduction potential by >300 mV relative to the non-protonated form. The ring-closed pyran form of the partially oxidized PDT may be protonated at the N-5 position by specific amino acids that hydrogen bond with the pterin ring. Since the presence of a pyran ring favors stronger electronic communication between the dithiolene chelate and the pterin, this leads to dramatic changes at the Mo ion that are reflected in the reduction potential. As such, we have described this behavior as a new type of ligand non-innocence [86,92,139], noting that various degrees of hydrogen bonding (i.e., remote charge effects) involving N-5 can lead to a variety of redox potential changes at the remote Mo ion that can be dynamic in nature.

**Figure 18:**
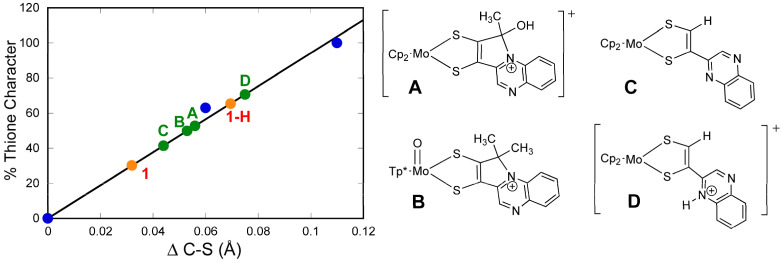
Correlation between the percent of thione-thiol resonance structure contribution to a series of model complexes, (**A**–**D**), **1**, and **1-H**. The straight line was derived from a best fit of the data for organic thiols, thiones, and NBO computations. Reproduced with permission from Ref. [92]. Gates, C.; Varnum, H.; Getty, C.; Loui, N.; Chen, J.; Kirk, M.L.; Yang, J.; Nieter Burgmayer, S.J. Protonation and Non-Innocent Ligand Behavior in Pyranopterin Dithiolene Molybdenum Com-plexes. *Inorg. Chem*. **2022**, *61*, 13728–13742. https://doi.org/10.1021/acs.inorgchem.2c01234. Copyright 2022 American Chemical Society.

Pterin protonation drives a large change in the electronic structure of the dithiolene chelate and the Mo environment. One indication of this change is the striking difference between the electronic absorption spectrum of **1-H** vs. **1** (Figure 17). Computational methods (DFT) allowed assignment of the intense 525 nm absorption to dithiolene → pterin intraligand charge transfer (ILCT) transitions from one-electron promotion between dithiolene π HOMO-1 and HOMO-2 orbitals to a pterin π* LUMO. Therefore, pterin protonation at N5 enhances the electron-deficient character of pterin and increases electron delocalization from dithiolene sulfurs to pterin. The electron density redistribution occurs from one specific dithiolene sulfur atom to the pterin and increases the percentage of the semi-oxidized thione-thiolate resonance structure. The amount of thione/thiolate resonance has been estimated at almost 70% based on the 0.066 Å difference in C-S bond lengths (thione C=S 1.784 Å and thiolate C-S 1.850 Å) from the DFT-optimized structures (Figure 18). The dominance of the thione/thiolate character in **1-H** is reflected in its stick drawing in Figure 15.

Overall, it is observed that protonation at the pterin N5 atom causes a significant increase in the asymmetry within the dithiolene chelate because the dithiolene is partially oxidized to a thione/thiolate. Since a thione/thiolate chelate is expected to be a poorer electron donor to the Mo atom, it can be anticipated that this change in the dithiolene unit will affect the Mo(V/IV) reduction potential, as observed by electrochemical measurements. Cyclic voltammetry of **1-H** shows a dramatic +315 mV positive shift of the Mo(V/IV) reduction potential when **1** is protonated to form **1-H**.

**Figure 19:**
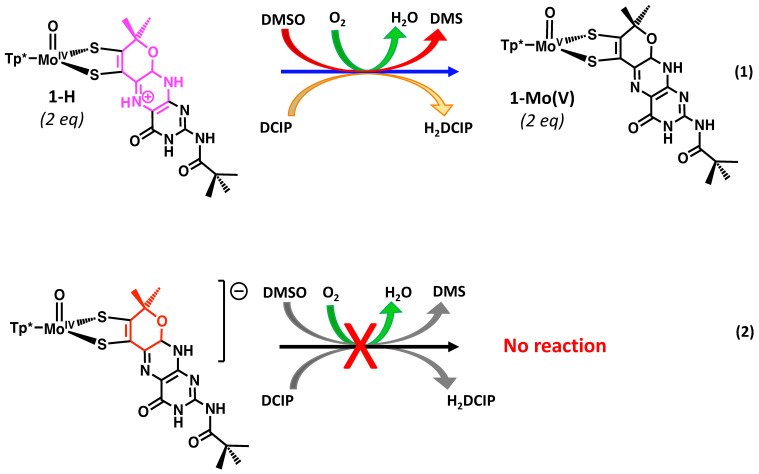
Protonated pyranopterin in **1-H** facilitates redox reactions (1) and (2) with DCIP, O_2_, and DMSO that do not occur in the absence of protonated pyranopterin.

Pyranopterin protonation also facilitates redox reactions between **1-H** and oxidants in reactions that do not occur in the absence of protonation of **1**. For example, **1-H** reacts immediately with the redox dye dichlorophenylindophenol (DCIP) and with air (O_2_), in contrast to **1,** which is unreactive to DCIP and is stable in air for more than 5 h. It is presumed that the pterin acts as a proton shuttle for these proton-dependent reactions, thereby lowering the energy for 2e^−^/2H^+^ redox processes (Figure 19). **1-H** also reacts sluggishly to reduce DMSO to DMS, whereas no reactivity between **1** and DMSO is observed (Figure 19).

#### 3.3.3. Contrasts between Pyranopterin and Pyranoquinoxaline Model Compounds

While the focus of this chapter is on the chemistry of a pyranopterin dithiolene ligand in model compounds, it is worth briefly addressing the differences observed between pterin- and quinoxaline-substituted dithiolenes. Quinoxaline is a simpler heterocycle that has been used in place of pterin for its greater solubility and ease of synthesis.

**Figure 20:**
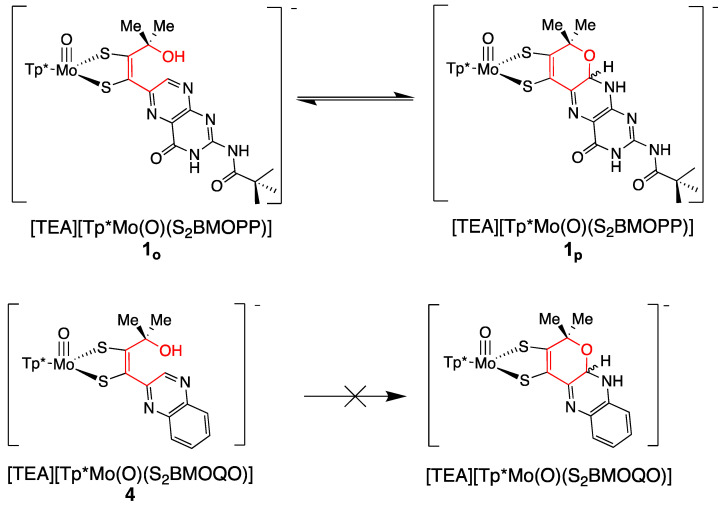
Comparison of pyran ring formation in pterin dithiolene and quinoxaline dithiolene compounds.

**Figure 21:**
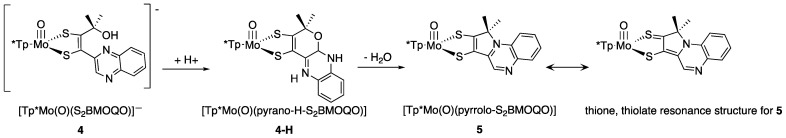
Protonation of 4 initiates pyran ring cyclization, forming unstable **4-H**, followed by -loss of -OH and intramolecular cyclization to a pyrroloquinoxaline compound **5**.

Two systems are presented to illustrate the stark differences between the chemistry observed on quinoxaline dithiolene vs. pterin dithiolene model complexes in terms of pyran ring behavior [51,92,111,112,113,133,139]. Figure 20 compares the chemistry of model compound **1** to its quinoxaline analog **4**. Reversible pyran ring formation is not observed for the quinoxaline dithiolene complex **4**. The absence of pyranoquinoxaline formation in **4** likely impacts the outcome of subsequent protonation reactions shown in Figure 21. The reaction of TFAA and **4** is signaled by an intense absorption at 505 nm, which has been interpreted as an initial protonation on quinoxaline at the N atom adjacent to the dithiolene chelate. This interpretation is based on the similarity to the 525 nm absorption of **1-H**. This initial protonated species **4-H** is unstable toward a competing reaction where protonation at the -OH of the side chain causes loss of water and an intramolecular cyclization to a pyrroloquinoxaline dithiolene ligand in **5**. It is notable that the Mo(V/IV) reduction potential is similar (±10 mV) for (open) non-pyranopterin dithiolenes bearing different side chains (C(OH)Me_2_, t-Bu, Ph, 2,4-F_2_Ph), indicating that there is little difference between a quinoxaline and a non-pyranopterin substituent on a dithiolene ligand coordinated to molybdenum. These data contrasts with the >50 mV shift in Mo(V/IV) reduction potential between the pyranopterin and open pterin in **1_p_** and **3** (Section 3.2.2).

**Figure 22:**
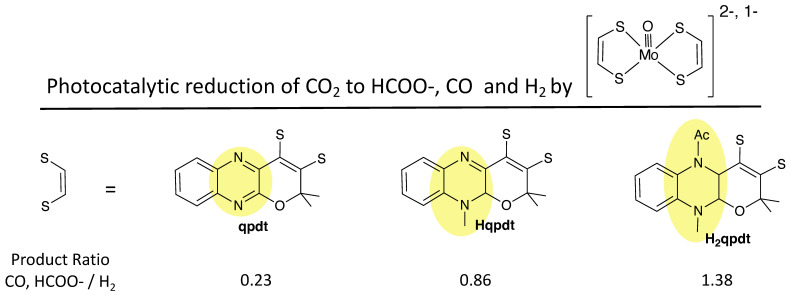
Bis-dithiolene oxo-Mo complexes with pyranoquinoxaline at different levels of reduction (oxidized, semi-reduced, reduced) photocatalyze reduction of H+ and CO_2_, where the proportion of reduced carbon products increases for the more reduced dithiolenes.

A second example illustrating differences between quinoxaline and pterin dithiolenes is from research reported by Fontecave and coworkers [142,143,144,145,146,147], who demonstrated that a pyranoquinoxaline dithiolene ligand (qpdt) can be synthesized where the quinoxaline is in its most oxidized form (Figure 22). By protecting the N atoms of the pyrazine ring, subsequent reduction of the quinoxaline to its semi- and fully reduced forms is possible, yielding the dithiolene ligands Hqpdt and H_2_qpdt. In the absence of N atom protection, no reduction of qpdt occurs, which contrasts the facile reduction of the pyranopterin in **1** to a reduced pyranopterin. Note that Hqpdt most closely resembles the pyranopterin structure of **1**, but it does not undergo any reversible pyran ring opening since the N10 methyl protection effectively prevents reversible pyran ring scission. Ligands qpdt, Hqpdt, and H_2_qpdt were used to make *bis*-dithiolene complexes of Mo(IV) and Mo(V), [MoO(qpdt)_2_]^2−^, [MoO(Hqpdt)_2_]^2−^, and [MoO(H_2_qpdt)_2_]^−^, whose structures model the *bis*-PDT-Mo active sites in formate dehydrogenase [142,144,147]. These three complexes were investigated as catalysts for CO_2_ reduction, and their ability to generate reduced products formate and CO vs. proton reduction to H_2_ is summarized in Figure 22. This work reveals that the reduced pyrazine ring in the quinoxalyl ligands is critical for determining the catalytic reactivity. For example, the Mo complex of qpdt is a more effective catalyst for proton reduction, whereas the H_2_qpdt complex favors photochemical catalysis of CO_2_ to HCOO- and CO.

## 4. What We Have Learned from Model Systems That Pertain to Moco in Enzymes? An Update

### 4.1. Previous Roles of the Pterin Defined

**Figure 23:**
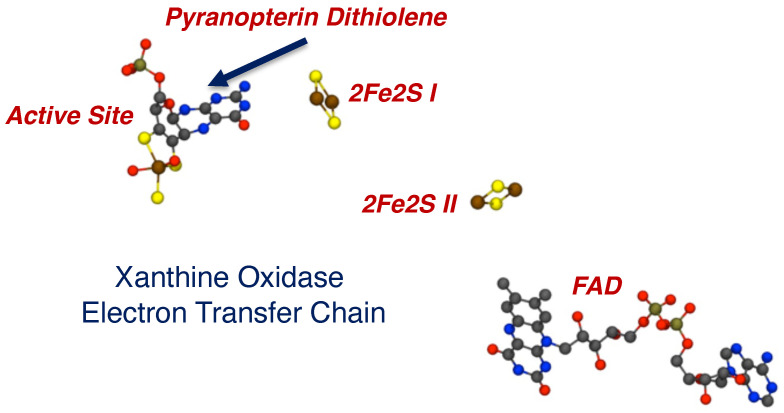
The electron transfer chain in XO, indicating a vectorial pathway for electron egress involving the Mo ion, the PDT, two spinach ferredoxin type 2Fe2S clusters, and a flavin. Electrons exit the enzyme at FAD.

The PDT is the least understood critical component of Moco in all pyranopterin Mo and W enzymes. This is remarkable, given the complex biosynthetic pathway and the ubiquitous nature of PDT in all the enzymes. At present, there is good evidence for three key roles of the PDT in catalysis, and these include functioning as an anchor for the Mo/W ion, serving as a through-bond electron transfer conduit for obligatory one-electron transfers in the electron transfer half reaction of the enzymes, and, with respect to the dithiolene component of the PDT, enabling redox potential modulation of the active site. Early protein crystallography studies indicated that the dithiolene component of the PDT could be either completely or partially dissociated from the metal ion, suggesting a role for this behavior in the catalytic cycles of some pyranopterin-containing enzymes. However, it is now understood that these structures represent active sites that have been damaged by the high flux of the X-ray beam during data collection. Thus, bidentate coordination of the dithiolene moiety is necessary for catalysis. Studies by Hille and coworkers were among the first to suggest a role of the PDT in electron transfer regeneration of the active site in xanthine oxidase (Figure 23) [148,149,150]. An extensive amount of model compound studies, including those detailed here, strongly suggest that the nature of the dithiolene ligand, remote charge effects, and the degree of the sulfur-fold angle can all affect the effective nuclear charge of the metal ion to drive large changes in the Mo redox potential. However, new roles for the PDT have been suggested that involve different oxidation states of the pterin component of the PDT, hydrogen bonding and proton transfer involving the pterin, and the role of thione-thiol resonance structure contributions to the electronic structure of the dithiolene chelate.

### 4.2. Recent Results Define New Roles for the PDT in Catalysis

The model studies described in Section 3 have provided specific examples of how the PDT ligand may contribute to catalysis in Mo enzymes. An early hypothesis that the PDT might serve to modulate the Mo redox potential has now been demonstrated and quantified for pterin-dithiolene model complexes. The partially reduced pyranopterin structure is electron withdrawing with respect to the Mo-dithiolene unit, and this results in the Mo(V) ion being a stronger oxidant in these systems. The redox-flexible dithiolene responds by accessing a partially oxidized thione/thiolate resonance structure. Pyran ring cleavage severs the PDT electron conduit, as the dithiolene is now electronically isolated from the pterin, which can now rotate out of the Mo-dithiolene plane. Reduction of pyranopterin is expected to also decrease this electronic relay from Mo-dithiolene to pterin as the sp^3^-hybridized bridgehead carbon between pterin and dithiolene interrupts extended π-conjugation in the PDT.

It is worth emphasizing that the degree of thione-thiolate character in the chelate ring is Mo oxidation state-specific, with thione-thiolate character being observed only for Mo(IV) [86,92,139]. Higher oxidation states of Mo typically result in the dithiol resonance form of the ligand dominating and an increase in the chelate ring S-S fold angle to reduce the effective nuclear charge on the Mo ion [79,88,91,122]. We have used the difference in dithiolene C-S bond distances Δ(C-S) as an indicator of the amount of thione/thiolate resonance contribution and, hence, pterin-dithiolene covalency [92]. The Mo(V) complex [Tp*MoO(S_2_BMOPP)] exhibits more equidistant C-S bonds (Δ(C-S) < 0.010 Å), and therefore a considerably less thione/thiolate resonance contribution is observed. This implies that a change in the Mo oxidation state provides a means of controlling the electronic structure of the Mo-dithiolene unit.

Considering the plethora of H-bonding interactions between protein amino acid residues in the PDT binding region and Moco (Figure 4), it seems likely that the pyranopterin component of the PDT may be involved in a proton relay [80] to facilitate proton-dependent redox processes. Our model studies reveal how pyranopterin protonation by acidic amino acids at the most basic N5 atom of the pterin can facilitate electronic communication between the pterin and the dithiolene chelate. However, this can only happen if the pyran ring is intact and not ring-opened. Conjugation favored by a closed pyran ring enforces co-planarity of the pterin and dithiolene, and this is key to tuning the basicity of the N5 position on the pterin. The strong effect of pyranopterin protonation on the Mo(5+/4+) reduction potential implies a coupling of pterin protonation and redox steps during catalysis. Hence, the presence of the pyranopterin dithiolene structure creates a proton-sensitive electronic switch with the potential to affect both the oxidative and reductive half reactions in the catalytic cycles of the enzymes.

Finally, a role for the semi-reduced pyranopterin form of PDT seems highly probable based on the entirety of the results from model studies. It cannot be overemphasized that it is only this semi-reduced pyranopterin form that accesses all the redox flexibility and variable Mo-S covalency in Moco that permits the effects of a reaction at one atom being transmitted throughout the entire Mo-pterin-dithiolene structure.

## 5. Outlook

We have shown that the semi-reduced PDT in Moco is expected to have a dramatic effect on the electronic structure of enzyme active sites, and we have spectroscopically characterized various complexes that display this inherent redox flexibility. Interestingly, there has not been any direct spectroscopic evidence of PDT redox in the enzymes to date [48]. Exciting early protein voltammetry studies that indicated oxygen atom transfer was coupled to the 2-electron redox activity of the PDT in MsrP (i.e., YedY) were later shown to be incorrect [151], with detailed spectroscopic studies on this system indicating the as isolated Mo(V) form derives from an enzyme that has been inhibited by the binding of an exogenous thiol [48]. However, given the propensity for redox activity involving the PDT and the spectroscopic signatures of semi-reduced PDTs in our model studies, we should carefully look for these enzyme forms in future studies. It is possible that semi-reduced pterins have not been observed because they are transient species in the catalytic cycles of specific pyranopterin Mo enzymes, or the protein functions in a novel way to inhibit this extraordinary redox flexibility.

## Data Availability

Data available in original cited literature and corresponding supporting information.
